# Supplementary material for: Adverse fetal birth outcomes and its associated factors among mothers with premature rupture of membrane in Amhara region, Ethiopia
Source: PLoS One. 2024 Apr 1;19(4):e0298319. doi: 10.1371/journal.pone.0298319 (PMC10984396; doi:10.1371/journal.pone.0298319)
Supplement: Attachment — Submitted filename: Adverse Birth Outcomes and Its Associated Factors Among Mothers with Premature Rupture of Membrane in Amhara Region, Ethiopiadocx.docx [file pone.0298319.s001.docx]

**Adverse Birth Outcomes and Its Associated Factors Among Mothers with Premature Rupture of Membrane in Amhara Region, Ethiopia**

**Summary of the Research**

Adverse birth outcomes are the leading cause of neonatal mortality worldwide. Ethiopia is one of the countries struggling to reduce neonatal mortality through different strategies, but neonatal mortality remains high for many reasons. Despite adverse birth outcomes beings public health problem in Ethiopia. This study aims to assess birth outcomes and associated factors among mothers with PROM at Specialized Hospitals in Amhara Region, Ethiopia. A retrospective institution-based cross-sectional study design was applied among 538 mothers with premature rapture of the membrane at Amhara region specialized hospitals. A simple random sampling technique was employed to select the medical charts diagnosed with PROM and giving birth in the hospital within the period from July 08, 2019, to July 07, 2021.The data was collected using a checklist, entered EPI Data version 3.1, and analyzed using SPSS version 23. A binary logistic regression model was used to see the association between independent and dependent variables. P-value <0.05 was used to declare its statistical significance. The AOR with 95% CI was used to measure the strength of the association. Adverse birth outcome among Premature rupture of membrane mothers was 33.1% [95% CI 29.2-37.2]. Rural residents [AOR=2.94, 95% CI:1.73-4.97], have a history of urinary tract infection [AOR=6.87, 95% CI: 2.77-17.01], anemia [AOR=7.51, 95% CI: 2.88- 19.62], previous history of adverse birth outcome [AOR=3.54, 95% CI: 1.32-9.47] and less than two years interpregnancy interval [AOR=6.07, 95% CI: 2.49- 14.77] were positively associated with adverse birth outcome compared to their counterparts.

**Areas for improvement**

**Title:**

Good title.

**Abstract:**

The authors should make sure that they use standard abbreviations in the manuscript. They should keep the abbreviations to a minimum. (I prefer to remove abbreviation if possible or mention the full form if you use it for the first time, then between brackets ( ), write the abbreviations, then continue with the same abbreviation).

The authors should revise the language to improve readability.

The authors should make sure that the abstract don’t exceed 300 words.

In the key words: please write factors as this term reflecting your study.

**Introduction:**

The authors should revise introduction section for grammar issues and language to improve readability.

PROM can cause stillbirth and other adverse birth outcomes due to umbilical cord prolapse and compression or placental abruption: please add references here.

The authors should join some related paragraphs together as it discuss the same concepts.

Ethiopia is one of the countries struggling to reduce neonatal mortality through different strategies. But still, neonatal mortality is remaining high: The authors should mention the details, especially the statistics, and should mention the importance of the study in order ensure that readers understand what the authors wanted to study.

The authors used statistics in the third and fourth paragraphs of introduction section, they should mention from where this statistics came, which mean from which country.

**Overall**

The authors need to arrange introduction section. Join the paragraphs and rewrite some paraphrase so, the readers will understand what you wanted to convey from messages. The authors should make sure that missing references are written within the introduction section. They also need to make sure to clearly mention the significant of your study. Also, please check grammar to make sure it is more readably for the readers.

**Material and Methods:**

**Study area,** the authors should delete any unnecessary information which are not indicative or important to know. The authors should mention the study site of this study only.

**Study design,** the authors should mention the important of the study design.

**Inclusion and exclusion criteria,** the authors should explain the inclusion and exclusion criteria of this study.

**Sample size calculation,** the authors should make sure that the sample size for this study is clear for the readers.

**Sampling procedures,** the authors should mention what exactly the what sampling techniques used in this study. Lottery is not sampling techniques.

**Dependent variable,** the authors should mention the dependent variable.

**Independent variables,** the authors should clarify independent variable.

**Data collection tool and measurement**, the authors should revise the language to improve readability. They should clearly mention data collection methods.

**Data quality control,** the authors should revise the language to improve readability.

**Data processing and analysis,** the authors should revise the language to improve readability.

**Results:**

The authors should follow appropriate labelling of the title of each table in the manuscripts according to the journal’s guidelines. They don’t have to include from July 08, 2019, to July 07, 2021, within the title of the tables. While the study appears to be sound, the language is unclear, making difficult to follow. Thus, the authors should work with a writing editor to improve readability of the text. The results section should be more clear to the readers to show the relationship between each independent variable with dependent variable.

**Discussion:**

However, the current finding is lower than the study conducted in Egypt, 61.3% (6) The lower adverse birth outcome in the current study might be a result of the higher number of smokers (10%) and teenage (15%) mothers in a study done in Egypt than in the current study: the authors should make this clear to the readers.

The authors should work with a writing editor to improve readability of the text.

The authors should arrange discussion section according the concepts appeared in results section.

**Conclusion:** no comments

**List of acronyms:** The authors should mention also what is COR.

**References:**

The authors should revise all references according to the guidelines provided.
