# Supplementary material for: Adverse fetal birth outcomes and its associated factors among mothers with premature rupture of membrane in Amhara region, Ethiopia
Source: PLoS One. 2024 Apr 1;19(4):e0298319. doi: 10.1371/journal.pone.0298319 (PMC10984396; doi:10.1371/journal.pone.0298319)
Supplement: Attachment — Submitted filename: Response to Reviewers.docx [file pone.0298319.s002.docx]

| S. N | Reviewer Comment | Author response |  |
| --- | --- | --- | --- |
|  | Reviewer #1 |  |  |
| 1 | There is no novelty most factors | Thank you for your comment! In our country context, neonatal death still increasing and it's challenging to reduce neonatal mortality. Recent studies help to provide recent evidence for policymaker and programmer. There is some variability from paper to prepare about the determinants of fetal adverse birth outcome, still it is not saturated. So, our study help to provide the recent evidence in the region as well as for the country. |  |
| 2 | Lacks clarity it’s not clear maternal or neonatal adverse outcome. | Thank you for this valuable comment, this study mainly done on Fetal adverse birth outcome. And we correct the title accordingly in the revised manuscript |  |
| 3 | It would have been good if the type of PROM specified. | Thank you for your comments. We include all forms of PROM, and we clearly state it in the revised manuscript. |  |
| 4 | Clearly put the magnitude of the problem, efforts done to reduce the impact, the identified gaps for those efforts and why your study useful among others. | Thank you for these important points, we address the comments in the revised manuscript. |  |
| 5 | I am not sure, the study called retrospective facility based cross sectional. | Thank you for your concern. We revised it “A Facility based Cross-sectional”. The study design is purely Cross-sectional; the word “retrospective” was stated to show the timing of event is in the past, but the data was collected at a point in time using secondary data. |  |
| 6 | Be consistent the number of specialized hospitals in the region. | Thank you for the comment, we correct it in the revised manuscript. |  |
| 7 | Include schematic presentation of sampling procedure. | Thank you for this interesting recommendation. We incorporate the schematic presentation of the sampling procedure in the revised manuscript. |  |
| 8 | Did you confident enough that stillbirth, prematurity, low birth weight and congenital anomalies are adverse birth outcome solely due to PROM. | Thank you for the questions. Basically, we can’t sure those adverse fetal birth outcomes are due to PROM alone, but we investigate the magnitude and it associated factors among those mothers. Because PROM is among the complication that have great impact on those fetal adverse outcomes, and the consequent neonatal and child growth and development.  To address this concern, in the introduction section of the revised manuscript; we add a statement that confirm “PROM is not the only causes for fetal adverse birth outcome, but it has a great impact on it”. |  |
| 9 | It would have been good if, validated tool was used for data collection. | Thank you for these comments. As we present in the method section of data collection tool and measurement section, we used a tool that was used by different articles in related contexts well as the global and national neonatal and maternal health related program documents and guidelines. Moreover, the tool was reviewed by four experts from the Amhara regional health bureau and Bahir Dar University. |  |
| 10 | No mechanism mentioned regarding encountering incomplete medical records. | Thank you for this interesting comments. We manage the incomplete medical recording by random replacement technique. This point is incorporated in the revised manuscript. |  |
| 11 | The independent variables are few, many intrapartum and postpartum variables are missed. | Thank you for your concern. We assess the independent variables that will have a relationship with the immediate adverse birth outcome as well as available in the patient charts. A total of 17 independent variables was checked their potential association with the dependent variable. |  |
| 12 | The reason why logistic regression was used justify using model fitness. | Thank you. We incorporate the important assumption of binary logistics regression in the revised manuscript. Basically, the model is fit with a Hosmer-Lemeshow model fit test, p>0.05. |  |
| 13 | Non respondent is not expected for chart review. | Thank you for your suggestion. We revised it in the revised manuscript. The incomplete medical cards were considered as non-respondents, but those incomplete cards were managed using random replacing technique. So, we wrongly present the replaced cards and now we address it per your comment. |  |
| 14 | The result of the first objective not presented separately. | Thank you for the comment. We present it in the revised manuscript. |  |
| 15 | Most of the literature used for discussions are not comparable with your study. |  |  |
|  | Reviewer #2 | | |
| 1. | The authors need to arrange the introduction section. Join the paragraph and rewrite some paraphrase, so the reader will understand what you wanted to convey from the message. | Thank you for these important comments and suggestion. We accordingly address all these point in the revised manuscript. |  |
| 2. | The author should make sure that missing references are written within the introduction section. | Thank you for you comment. We cited all missing references. |  |
| 3. | They also need to make sure to clearly mention the significance of your study. | Thank you for these important. We clearly mention the study significance on the last paragraph of the introduction in the revised manuscript. |  |
| 4. | Check grammar to make sure it is more readably for the readers. | Thank you for your suggestion, we checked the grammar and its readability by using online grammar checker and frequent expert reading and correct. |  |
