# Supplementary material for: Adverse fetal birth outcomes and its associated factors among mothers with premature rupture of membrane in Amhara region, Ethiopia
Source: PLoS One. 2024 Apr 1;19(4):e0298319. doi: 10.1371/journal.pone.0298319 (PMC10984396; doi:10.1371/journal.pone.0298319)
Supplement: Attachment — Submitted filename: feedback PONE adverse fetal birth review 2.docx [file pone.0298319.s003.docx]

**Adverse Fetal Birth Outcomes and Its Associated Factors among Mothers with Premature Rupture of Membrane in Amhara Region, Ethiopia** **(Second Review)**

| **Review 1** | **Authors** | **Comment of reviewer** |
| --- | --- | --- |
| The authors need to arrange the introduction section. Join the paragraph and rewrite some paraphrase, so the reader will understand what you wanted to convey from the message | Thank you for these important comments and suggestion. We accordingly address all these point in the revised manuscript. | Changes had been done, thank you |
| The author should make sure that missing references are written within the introduction section. | Thank you for your comment. We cited all missing references. | Changes had been done, thank you |
| They also need to make sure to clearly mention the significance of your study. | Thank you for these important. We clearly mention the study significance on the last paragraph of the introduction in the revised manuscript | Well done, Changes had been done, thank you |
| Check grammar to make sure it is more readably for the readers | Thank you for your suggestion, we checked the grammar and its readability by using online grammar checker and frequent expert reading and correct. | Changes had been done, thank you |
