# Supplementary material for: Adverse fetal birth outcomes and its associated factors among mothers with premature rupture of membrane in Amhara region, Ethiopia
Source: PLoS One. 2024 Apr 1;19(4):e0298319. doi: 10.1371/journal.pone.0298319 (PMC10984396; doi:10.1371/journal.pone.0298319)
Supplement: Attachment — Submitted filename: Response to Reviewers .docx [file pone.0298319.s004.docx]

| S. N | Reviewer Comment | Author response |  |
| --- | --- | --- | --- |
|  | Reviewer #3 |  |  |
| 1 | Still the availability of data from client charts is under question since it is secondary data. Since the documentation of client charts in Ethiopia is knowingly poor. Is ethnicity documented in the client chart? Is only replacing mechanism can reduce the bias due to incomplete information? | Thank you for your comment!   - In the patient individual folder, there is no question about ethnicity, but there is a place that requests the current address of the patient so, we accept your comment, and we correct the word by region of residency instead of ethnicity. - Regarding your second concern of managing missing data, The issue is very interesting; at the proposal stage, We planned to manage missing data other than our outcome interest using “Average Imputation” and our outcome interest as already stated in the manuscript managed using a replacement mechanism. But, we didn’t face any missing data/information. The missing data was found in the outcome variable only. |  |
| 2 | Why pre-test in the same hospital? | Thank you for this valuable comment, The purpose of the pretest is to check the availability of variables on the chart and to deal with how to address these issues before data collection begins. In our study nature, there is no risk of information Contamination, since it is a chart review. So, we tried to follow the principle of the same modalities of pre-test and main data collection. |  |
| 3 | The discussion section needed some rewriting by using good research discussion section writing steps: - Step 1: Summarize your key findings. Start this section by reiterating your research problem and concisely summarizing your major findings. ...Step 2: Give your interpretations. ...Step 3: Discuss the implications. ...Step 4: Acknowledge the limitations. ...Step 5: Share your recommendations. | Thank you for these important points, we address the comments as per your recommendation on the revised manuscript. |  |
| 4 | The manuscript needs some English grammar corrections. | Thank you for your suggestion, we checked the grammar and its readability by using an online grammar checker and frequent expert reading and correcting it accordingly. |  |
